# Mapping Mammalian Cell-type-specific Transcriptional Regulatory Networks Using KD-CAGE and ChIP-seq Data in the TC-YIK Cell Line

**DOI:** 10.3389/fgene.2015.00331

**Published:** 2015-11-18

**Authors:** Marina Lizio, Yuri Ishizu, Masayoshi Itoh, Timo Lassmann, Akira Hasegawa, Atsutaka Kubosaki, Jessica Severin, Hideya Kawaji, Yukio Nakamura, FANTOM consortium, Harukazu Suzuki, Yoshihide Hayashizaki, Piero Carninci, Alistair R. R. Forrest

**Affiliations:** ^1^RIKEN Center for Life Science TechnologiesYokohama, Japan; ^2^Division of Genomic Technologies, RIKEN Center for Life Science TechnologiesYokohama, Japan; ^3^RIKEN Preventive Medicine and Diagnosis Innovation ProgramYokohama, Japan; ^4^Telethon Kids Institute, The University of Western AustraliaSubiaco, WA, Australia; ^5^Cell Engineering Division, RIKEN BioResource CenterIbaraki, Japan; ^6^QEII Medical Centre and Centre for Medical Research, Harry Perkins Institute of Medical Research, The University of Western AustraliaNedlands, WA, Australia

**Keywords:** ChIP-seq, transcriptional regulatory network, perturbation, pancreas, CAGE, FANTOM5

## Abstract

Mammals are composed of hundreds of different cell types with specialized functions. Each of these cellular phenotypes are controlled by different combinations of transcription factors. Using a human non islet cell insulinoma cell line (TC-YIK) which expresses insulin and the majority of known pancreatic beta cell specific genes as an example, we describe a general approach to identify key cell-type-specific transcription factors (TFs) and their direct and indirect targets. By ranking all human TFs by their level of enriched expression in TC-YIK relative to a broad collection of samples (FANTOM5), we confirmed known key regulators of pancreatic function and development. Systematic siRNA mediated perturbation of these TFs followed by qRT-PCR revealed their interconnections with *NEUROD1* at the top of the regulation hierarchy and its depletion drastically reducing insulin levels. For 15 of the TF knock-downs (KD), we then used Cap Analysis of Gene Expression (CAGE) to identify thousands of their targets genome-wide (KD-CAGE). The data confirm *NEUROD1* as a key positive regulator in the transcriptional regulatory network (TRN), and *ISL1*, and *PROX1* as antagonists. As a complimentary approach we used ChIP-seq on four of these factors to identify *NEUROD1, LMX1A, PAX6*, and *RFX6* binding sites in the human genome. Examining the overlap between genes perturbed in the KD-CAGE experiments and genes with a ChIP-seq peak within 50 kb of their promoter, we identified direct transcriptional targets of these TFs. Integration of KD-CAGE and ChIP-seq data shows that both *NEUROD1* and *LMX1A* work as the main transcriptional activators. In the core TRN (i.e., TF-TF only), *NEUROD1* directly transcriptionally activates the pancreatic TFs *HSF4, INSM1, MLXIPL, MYT1, NKX6-3, ONECUT2, PAX4, PROX1, RFX6, ST18, DACH1*, and *SHOX2*, while *LMX1A* directly transcriptionally activates *DACH1, SHOX2, PAX6*, and *PDX1*. Analysis of these complementary datasets suggests the need for caution in interpreting ChIP-seq datasets. (1) A large fraction of binding sites are at distal enhancer sites and cannot be directly associated to their targets, without chromatin conformation data. (2) Many peaks may be non-functional: even when there is a peak at a promoter, the expression of the gene may not be affected in the matching perturbation experiment.

## Introduction

Regulation of gene expression by combinations of transcription factors (TFs) is a fundamental process that determines cellular identity and functions. TFs have the ability to recognize and bind short sequence motifs throughout the genome, and, either alone or in combination with other TFs, modulate mRNA levels in a cell until it acquires the predetermined phenotype (Mitchell and Tjian, 1989; Wray et al., 2003). In humans it has been estimated that there are at least 411 different cell types (Vickaryous and Hall, 2006) and 1500–2000 different transcription factors (Roach et al., 2007; Vaquerizas et al., 2009; Wingender et al., 2015), with ~430 TFs expressed at appreciable levels in any given primary cell type (Forrest et al., 2014). Identifying key cell type specific transcription factors and their targets is fundamental to understanding cellular states, and is important for regenerative medicine where efforts are made to direct differentiation of stem cells toward a medically relevant cell type (Cahan et al., 2014).

Over the years, multiple approaches to map the targets of TFs have been developed. Computational approaches that predict TF targets based upon their co-expression with a given TF and/or the presence of a transcription factor binding site motif (TFBS) in their promoter regions have helped to identify direct targets (Wasserman and Sandelin, 2004; Tompa et al., 2005; Valouev et al., 2008; FANTOM Consortium et al., 2009); however, these are purely predictive methods and the validation rate, when experimental validations are carried out, is low. Motif prediction methods are limited as the vast majority of our TFs have no well-defined TFBS, and TFs from the same family bind very similar motifs. Even for those cases where a motif is known, the information content is so low that the majority of binding site predictions will likely be false positives (Wasserman and Sandelin, 2004). Lastly, unless the expression levels of the TFs themselves are taken into consideration, inaccurate predictions can be made where a binding event may be predicted as important despite the fact that the corresponding TF is not even present in the cell.

Alternatively, TF targets can be identified experimentally. Experimental perturbation of TFs (Hilger-Eversheim et al., 2000) followed by expression profiling can identify global sets of genes affected by the given TF. This is a powerful approach, but does not discriminate direct from indirect targets (genes regulated by TFs which are regulated by the perturbed TF). Another experimental approach directly determines physical binding sites in the genome using protocols such as ChIP-CHIP, DamID or ChIP-seq (van Steensel and Henikoff, 2000; Horak et al., 2002; Robertson et al., 2007). The caveat with these methods lies in that they do not distinguish functional from non-functional binding. By combining the perturbation and physical interaction approaches we can overcome the limitations of each.

The remaining issue, however, is the scale of the problem. TF-target interactions vary between cell types as there are different combinations of transcription factors expressed and different chromatin configurations in each cell type. Thus, ultimately, what we need is a compendium of cell type specific regulatory networks for every cell type that makes up the human body. Given its scale, the problem necessitates prioritization of the cell type to be studied and the sets of TFs considered. We need ways to identify which TFs are most important to a given cell type.

Recently, the FANTOM5 project used single molecule sequencing to generate CAGE (Kanamori-Katayama et al., 2011) across a large collection of human and mouse primary cells, cell lines and tissue samples, providing a nearly comprehensive set of human and mouse, promoter and enhancer regions and their expression profiles (Andersson et al., 2014; Forrest et al., 2014). Importantly, for the prioritization of key TFs, the FANTOM5 CAGE data boasts expression profiles for 94% (1665/1762) of human TFs; this can be used to generate cell-type-specific ranked lists (expression relative to median across almost 1000 samples). What emerged from those lists is that the TFs with the most enriched expression in a given primary cell type often had phenotypes relevant to that cell type [e.g., mutations of osteoblast enriched TFs resulted in bone phenotypes, hematopoietic stem cell enriched TFs in blood phenotypes and inner ear hair cell enriched TFs in deafness (Forrest et al., 2014)]. These enriched TFs are therefore likely key components of cell-type-specific transcriptional regulatory networks (TRNs). To probe cell type enriched TFs in more detail, we explored an integrated approach for dissecting TRNs using siRNA knock-down, qRT-PCR, CAGE (Shiraki et al., 2003), and ChIP-seq (Robertson et al., 2007).

The large numbers of cells required for our systematic studies made it necessary to find an easily expandable cell line. Reviewing the FANTOM5 expression profiles, we chose an interesting cell line, TC-YIK (Ichimura et al., 1991), derived from an argyrophilic small cell carcinoma (ASCC) of the uterine cervix, which expresses insulin and showed enriched expression for dozens of pancreatic transcription factors. We show that TC-YIK cells express 75% of a set of genes previously reported as islet cell specific and 85% of a set of genes previously reported as beta cell specific. Given the difficulty in obtaining primary human beta cells for research, our results may be of interest to studying pancreatic transcriptional regulation, with the caveat that we are only using TC-YIK as an experimentally tractable cell line model to examine the prediction of key TFs; it is a non-islet-cell insulinoma and therefore the regulatory edges inferred here may not generalize to primary islet cells.

**Figure 1:**
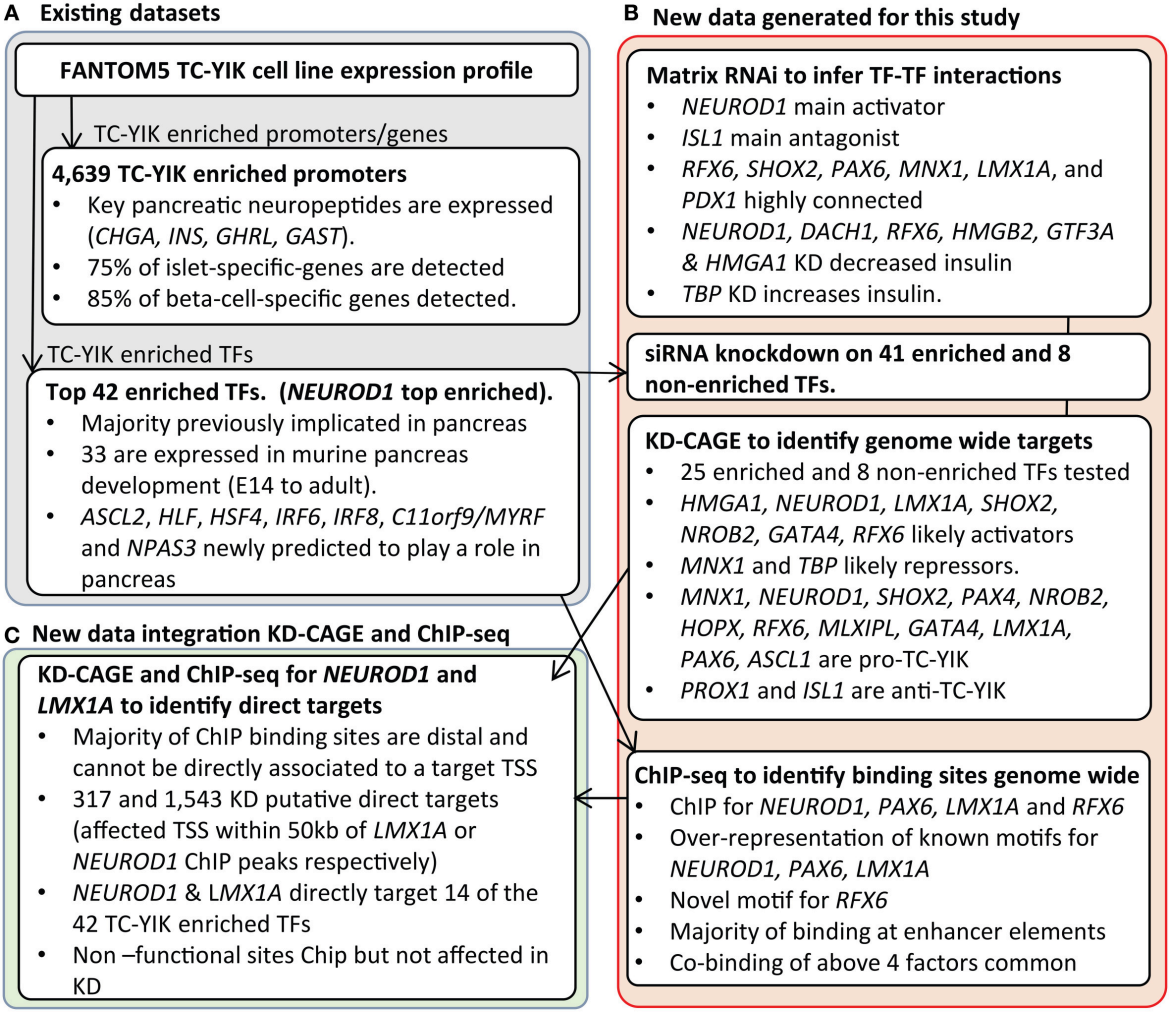
**Diagram showing the workflow of the analyses. (A)** The FANTOM5 data reveal the TC-YIK cell line expresses key pancreatic neuropeptides and pancreatic transcription factors. **(B)** New data is generated for this study including siRNA perturbation of TC-YIK enriched and non-enriched TFs. siRNA perturbed samples are profiled by qRTPCR in a matrix RNAi design and by CAGE to globally identify target promoters. ChIP-seq on 4 key TFs is also carried out to identify genome wide binding sites. **(C)** KD-CAGE and ChIP-seq data are integrated to identify directly regulated targets of *NEUROD1* and *LMX1A*.

Using newly created genome-wide datasets on TC-YIK enriched TFs, and a comparative set of non-enriched TFs, we sought to determine the importance of each factor in maintaining the TC-YIK cell state. Knock-down followed by CAGE profiling allowed us to identify, genome-wide, the set of genes affected by each TF, while integration with ChIP-seq data on the same factors allowed us to further discriminate direct from indirect TF targets. We present the results of the TC-YIK analysis and show that the combination of CAGE and ChIP-seq on key TFs is a powerful approach for studying mammalian transcriptional networks and necessary for dissection of direct and indirect edges. An overview of the datasets used, our analysis and the main findings are summarized in the workflow shown in Figure 1.

This work is part of the FANTOM5 project. Data download, genomic tools and co-published manuscripts have been summarized at http://fantom.gsc.riken.jp/5/.

## Results

### The TC-YIK cell line expresses pancreatic islet cell transcripts

Previously, TC-YIK cells were shown to generate neurosecretory granules and express chromogranin A (*CHGA*) and gastrin (*GAST*; Ichimura et al., 1991). A systematic review of endocrine hormones and peptides detected in TC-YIK confirmed *CHGA* and *GAST* were expressed at high levels and revealed also expression of insulin (*INS*), ghrelin (*GHRL*), and transthyretin (*TTR*; Table 1). All of these proteins [insulin, gastrin (*GAST*; Wang et al., 1993; Rooman et al., 2002; Téllez et al., 2011), ghrelin [(*GHRL*; Date et al., 2002; Wang et al., 2008; Arnes et al., 2012), transthyretin (*TTR*; Refai et al., 2005; Su et al., 2012), and chromogranin A (*CHGA*, a precursor of pancreatic chromostatin; Cetin et al., 1993)] play key roles in the pancreas (Table 1). In contrast to insulin, which is a biomarker for pancreatic beta cells, somatostatin (*SST*), glucagon (*GCG*), and islet amyloid polypeptide (*IAPP*), the biomarkers for pancreatic delta, alpha, and gamma cells, respectively, were lowly expressed or absent in TC-YIK cells. We next examined the expression of genes described in the beta cell gene atlas (Kutlu et al., 2009) as being specifically expressed in human islets. We find that 75% of the 938 human islet tissue specific genes reported by the authors are detected in TC-YIK [Supplementary Table 1, ≥ 5 tags per million (TPM)]. The authors provide a further subset of 445 genes that are enriched in alpha and/or beta cells and overlap the islet specific list (76 are expressed > 2-fold higher in alpha cells and 153 are expressed > 2-fold higher in beta cells). In TC-YIK, we find that 65% of these alpha cell enriched genes and 85% of the beta cell enriched genes are detected (Supplementary Table 2, ≥ 5 TPM). From this review we conclude that, although TC-YIK does not completely recapitulate the beta cell transcriptome, it shares significant similarity to islet cells. For this reason TC-YIK is sufficiently interesting for the purposes of an investigative study integrating CAGE and ChIP-seq data. Lastly, although there are rare reports of non-islet-cell insulinomas that ectopically express insulin [e.g., kidney (Ramkumar et al., 2014), liver (Furrer et al., 2001), brain (Nakamura et al., 2001)] and additional cases of argyrophilic small cell carcinoma (ASCC) of cervix (Kiang et al., 1973; Seckl et al., 1999), ours is the first report to our knowledge that identifies a non-islet-cell line (TC-YIK) where the majority of the beta cell program is active.

**Table 1:** **Neurosecretory peptide expression in TC-YIK**.

**Gene**	**Expression in FANTOM5 (TPM)**			
	**TC-YIK**	**Rank (out of 988 samples)**	**Max**	**Sample expressing highest level of peptide**
*CHGA*	6062.51	1	6062.51	TC-YIK
*TTR*	1202.73	21	60441.3	medulla oblongata, adult
*GAST*	1096.66	1	1096.66	TC-YIK
*INS*	50.13	4	5119.98	Duodenum, fetal
*GHRL*	15.37	5	54.13	Eosinophils
*SST*	7.81	93	3612.79	Duodenum, fetal
*IAPP*	0	NA	26.58	Pancreas, adult
*GCG*	0	NA	3534.95	Gastric cancer cell line AZ521

### Pancreatic transcription factors are enriched in TC-YIK cells

To identify TC-YIK-enriched-transcription factors, we ranked all 1665 human TFs according to their expression in TC-YIK cells relative to the median expression across the 988 human samples in the FANTOM5 phase 1 collection (Forrest et al., 2014). The highest ranked TF was *NEUROD1*, a factor known to be key in the differentiation of beta cells and insulin production (Itkin-Ansari et al., 2005; Guo et al., 2012). Furthermore, of the 42 most TC-YIK enriched TFs (enrichment score > 1.25, ~18-fold enrichment over median expression levels), 33 were previously implicated in pancreatic biology, including direct regulators of insulin (Sander and German, 1997), key factors for islet cell development (Wang et al., 2005; Guo et al., 2011), genes associated with diabetes (Foti et al., 2005) and with pancreatic endocrine tumors (Johansson et al., 2008; Table 2, Supplementary Table 3).

**Table 2:** **TFs enriched in TC-YIK and their putative function in pancreas**.

**TF_symbol**	**Expression TPM**	**Enrichment log10 (TC-YIK+1/median+1)**	**Insulin or pancreatic biology?**	**Detected in mouse developing pancreas**	**Experiments**
**TRANSCRIPTION FACTORS WITH ENRICHED EXPRESSION IN TC-YIK CELLS**					
*NEUROD1*	593	2.77	Yes	Yes	Si, CA, CS
*INSM1*	519	2.72	Yes	Yes	–
*PAX6*	296	2.47	Yes	Yes	Si, CA, CS
*NKX6-3*	239	2.38	Yes	No	–
*ARX*	237	2.38	Yes	Yes	Si
*MLXIPL*	218	2.34	Yes	Yes	Si, CA
*RFX6*	146	2.17	Yes	Yes	Si, CA, CS
*ONECUT2*	151	2.14	Yes	Yes	Si, CA
*PAX4*	133	2.13	Yes	Yes	Si, CA
*PDX1*	127	2.11	Yes	Yes	Si
*DACH1*	269	2.05	Yes	Yes	Si, CA
*ISL1*	102	2.01	Yes	Yes	Si, CA, CS
*FEV*	94	1.98	Yes	No	Si
*HOPX*	168	1.95	Yes	Yes	Si, CA
*FOXA2*	88	1.95	Yes	Yes	Si
*ST18*	78	1.90	Yes	Yes	–
*HNF4G*	75	1.88	Yes	Yes	–
*PROX1*	106	1.84	Yes	Yes	Si, CA
*HNF4A*	69	1.84	Yes	Yes	Si
*ELF3*	51	1.71	Yes	Yes	Si
*SHOX2*	62	1.70	Yes	No	Si, CA
*NPAS3*	55	1.63	No	Yes	–
*CDX2*	41	1.63	Yes	Yes	–
*HOXA10*	40	1.61	Yes	No	Si
*MNX1*	38	1.59	Yes	Yes	Si, CA
*ASCL2*	34	1.54	No	Yes	–
*TFAP2A*	97	1.53	Yes	No	–
*IRF8*	31	1.51	No	Yes	Si
*CASZ1*	70	1.51	Yes	Yes	–
*SIX3*	30	1.49	No	No	Si
*C11orf9/MYRF*	62	1.49	No	Yes	–
*MYT1*	26	1.43	Yes	Yes	Si
*HOXB13*	26	1.43	Yes	No	Si
*ASCL1*	25	1.42	Yes	Yes	Si, CA
*NR0B2*	24	1.41	Yes	Yes	Si
*LMX1A*	24	1.40	Yes	No	Si, CA, CS
*HSF4*	27	1.33	No	Yes	–
*HES6*	71	1.32	Yes	Yes	–
*HLF*	23	1.31	No	Yes	Si
*IRF6*	23	1.30	No	Yes	–
*DLX6*	19	1.29	No	No	Si
*GATA4*	18	1.28	Yes	Yes	Si, CA
**UBIQUITOUS TRANSCRIPTION FACTORS EXPRESSED IN TC-YIK BUT NOT ENRICHED**					
*ATF5*	290	0.73	No	Yes	Si, CA
*HMGB2*	243	0.37	No	Yes	Si, CA
*GTF3A*	213	0.36	No	Yes	Si, CA
*HMGA1*	672	0.34	Yes	Yes	Si, CA
*TBP*	29	0.15	No	Yes	Si, CA
*TAF9*	80	0.09	No	Yes	Si, CA
*TCF25*	90	−0.10	No	Yes	Si, CA
*TAF10*	75	−0.33	No	Yes	Si, CA

*An extended version of the table is provided as Supplementary Table 3 with references to pancreatic biology. Experiments used in this paper (Si, siRNA perturbation; CA, cap analysis of gene expression; CS, ChIP-seq). TC-YIK enriched factors that were not tested by siRNA were excluded due to oligo design or knock-down efficiency problems*.

CAGE profiling of the mouse orthologs throughout pancreatic development (also profiled in FANTOM5) detected 33 of the 42 TFs in at least one stage with most changing expression levels over time (Supplementary Figure 1). This added support for a further seven of the remaining nine TFs enriched in TC-YIK (*ASCL2, HLF, HSF4, IRF6, IRF8, MYRF*, and *NPAS3*) as likely important factors in pancreatic development.

### Assessing the interconnection of key TFs

A key question is whether the cell type enriched TFs identified in FANTOM5 are key regulators of the cellular state and whether these enriched factors are more (or less) important than housekeeping TFs that are more broadly expressed. Logic would suggest that those TFs expressed in an enriched manner are more likely to be regulated by other enriched TFs, and that their targets are also more likely to be enriched. To test our assumption, we first carried out siRNA perturbation of a set of enriched and non-enriched (but expressed) TFs in TC-YIK cells and assessed their effect on expression of enriched and non-enriched targets by qRT-PCR.

Multiple siRNAs were tested for each enriched factor and the one with the best efficiency was kept; siRNAs for 26 TFs reduced expression below 50%, a further 7 suboptimal siRNAs reduced expression to 51–77% of that of the scrambled control, while for the remaining TFs we were unable to find an efficient siRNA (Supplementary Table 4). An additional 8 non-enriched TFs were also perturbed below 50% (Table 2). After perturbation, RNA was extracted and qRT-PCR was used to measure the knock-down response in a 41 × 52 matrix of expression changes, where 41 columns represent the TFs that were perturbed and 52 rows represent the measured qRT-PCR values of target genes after perturbation (Supplementary Table 5). Experiments were carried out in triplicate and knock-down was assessed relative to a scrambled siRNA sequence. Of the ~2000 potential (TF-target) edges tested, 551 were up- or down-regulated 1.5-fold or more [threshold as used in our previous studies (Tomaru et al., 2009)].

Looking at the number of affected targets for each TF knock-down (out degree) and the number of knock-downs that affected each TF (in degree; summarized in Supplementary Table 6) we identified *NEUROD1* as a key activator at the top of the hierarchy. *NEUROD1* knock-down caused down-regulation of 21 of the 52 tested targets (the most influenced being *PAX4*, followed by *GHRL, INS, GAST, CHGA, GCK, RFX6*, and *PAX6*). In an analogous way, *ISL1* was the main antagonist in the network, where its knock-down affected 11 targets, all of which were up-regulated (among those *CHGA, LMX1A, PAX4*, and *NEUROD1*). Other likely key TFs, *RFX6, SHOX2, PAX6, MNX1, LMX1A*, and *PDX1* also strongly affected several targets.

**Figure 2:**
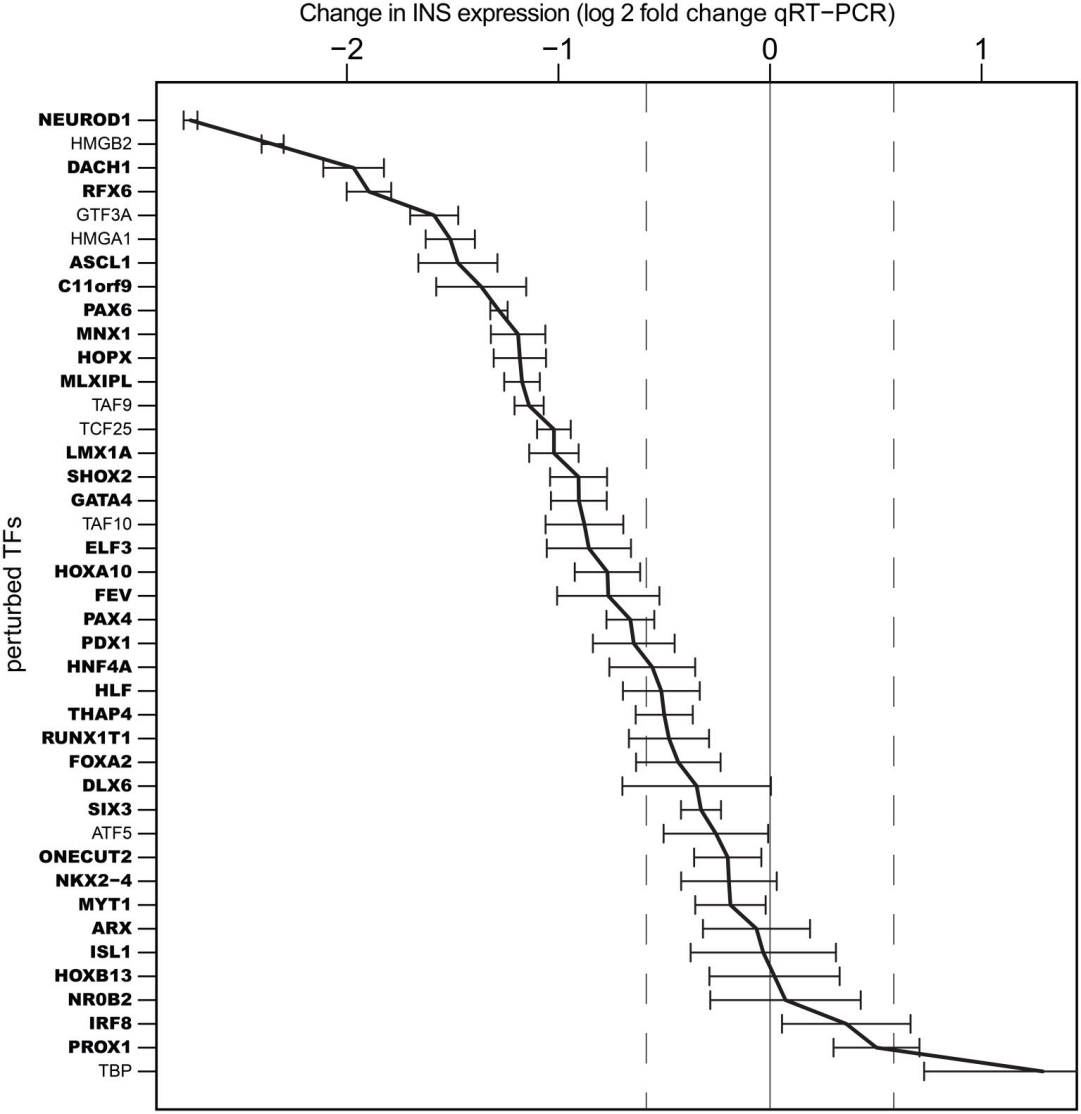
**Influence of transcription factor knock-down on ***INS*** expression**. Log2 expression fold changes for *INS* gene upon siRNA perturbation of 41 TFs. *NEUROD1* knock-down caused the most down–regulation of insulin expression, while highest up-regulation was observed in *TBP* knock-down. Error bars indicate standard deviation over triplicate measurements. TFs in bold indicate those that were TC-YIK-enriched rather than ubiquitous.

Of note, knock-down of 28 of the 33 TFs enriched in TC-YIK and 7 of the 8 non-enriched TFs affected insulin expression levels, with the enriched factors *NEUROD1, DACH1, RFX6*, and the non-enriched TFs *HMGB2, GTF3A*, and *HMGA1* knock-down causing the greatest decreases in insulin transcript levels (Figure 2). Interestingly, knock-down of the non-enriched TF TATA binding protein (*TBP*) led to the highest increase in insulin transcript, which may indicate a shift in the balance between TATA dependent and TATA independent transcription.

### Identifying genome-wide TF targets using knock-down and cage

The above section focused on a limited and biased set of 52 target transcripts. We next applied CAGE [KD-CAGE; (Vitezic et al., 2010)] to identify genome-wide the sets of promoters that were perturbed after knock-down of 15 of the enriched TFs and all 8 non-enriched TFs using the same RNA samples as used in the qRT-PCR. Notably the fold changes observed by CAGE and qRT-PCR were highly correlated (Supplementary Figure 2), indicating the suitability of CAGE for this experiment.

**Figure 3:**
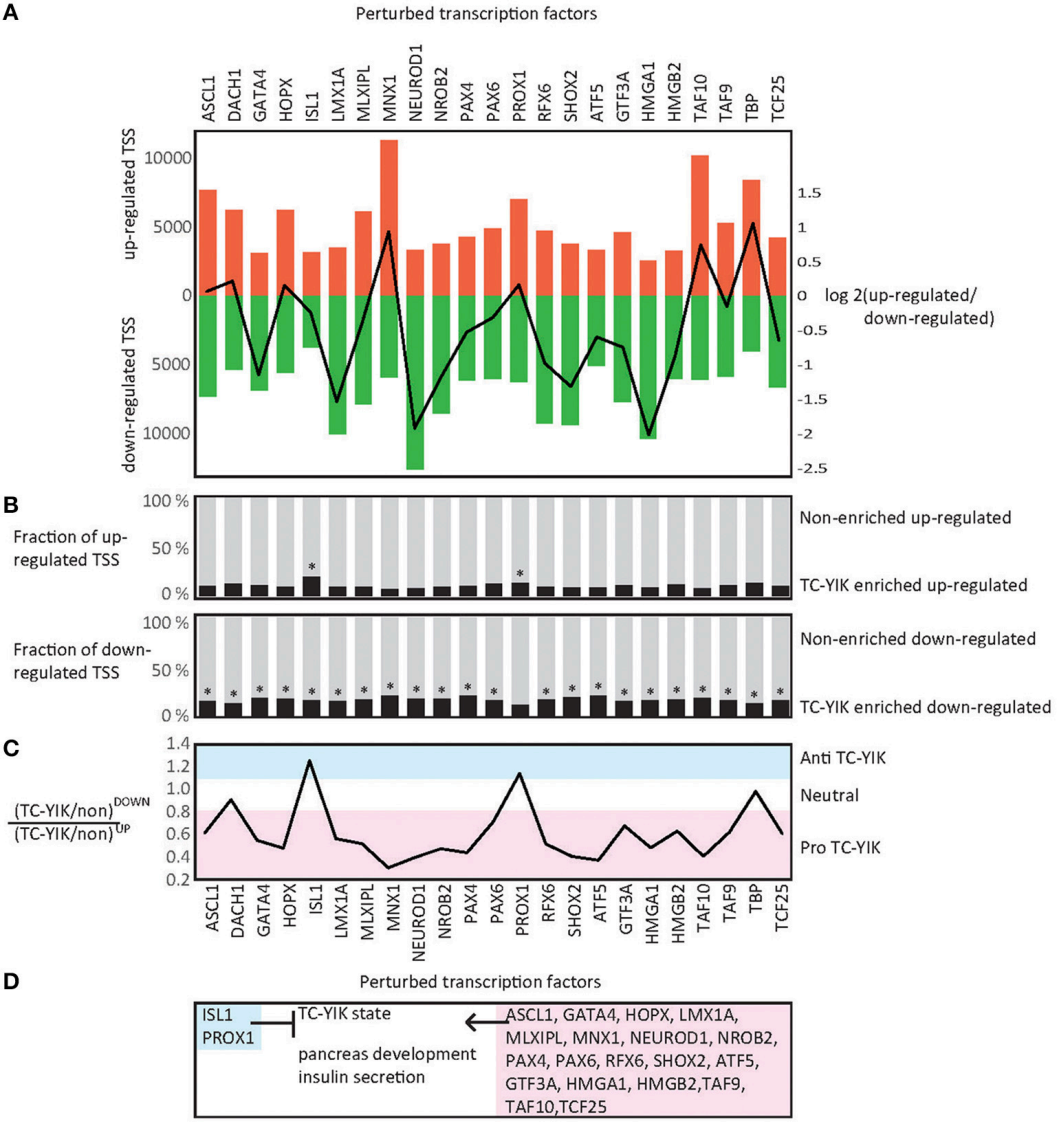
**KD-CAGE analysis. (A)** Up-regulated and down-regulated TSSs in KD-CAGE experiments. Bars indicate, the numbers of up-regulated, and down-regulated TSSs detected by edgeR (*p* < 0.05) after siRNA knockdown of each factor. Line indicates the log transformed ratio of up-regulated to down-regulated TSS (e.g., note *NEUROD1* causes a much larger number of down-regulated TSS than up-regulated ones, while *MNX1* shows the reverse). **(B)** Fractions of up or down-regulated promoters that are TC-YIK-enriched or non-enriched. **(C)** Comparison of the ratios of TC-YIK-enriched to non-enriched promoters for up and down-regulated TSS sets. Note, *ISL1* and *PROX1* appear antagonistic to the TC-YIK state. **(D)** Diagram summarizing the results of the state enrichment and gene ontology enrichment analyses. ^*^Indicates at least 15% of the up or down-regulated promoters were TC-YIK enriched.

Promoters specifically affected by the TF knock-downs in comparison to scrambled siRNA control samples were then identified using edgeR (Robinson et al., 2010; Supplementary Table 7). Similar numbers of affected promoters were detected for enriched and non-enriched TFs; between 8229 and 19,467 and between 9922 and 18,362 promoters respectively (Supplementary Table 8). For six of the TF knock-downs (*HMGA1, NEUROD1, LMX1A, SHOX2, NROB2, GATA4, RFX6*), there were at least twice as many down-regulated promoters as up-regulated ones, suggesting that these factors work as activators. Conversely, for knock-down of *MNX1* and *TBP* we observed at least twice as many up-regulated promoters as down-regulated ones, suggesting they work as repressors (Figure 3A).

### Identifying TFs important for maintaining cell state

To understand which TFs are responsible for maintaining the TC-YIK cell state, we next identified a set of 4639 promoters with enriched expression (>3-fold) in TC-YIK compared to median expression in FANTOM5. We refer to this set as TC-YIK-enriched-promoters, and to the remainder as non-enriched-promoters. We then used these sets to separate TFs into synergists or antagonists to the cell fate: if perturbation of a TF causes down-regulation of a significantly larger fraction of TC-YIK-enriched-promoters than non-enriched-promoters, then this would suggest that the factor in question is important for maintaining the TC-YIK state (pro-TC-YIK); similarly, if the perturbation led to up-regulation of a significantly larger fraction of TC-YIK-enriched-promoters than non-enriched-promoters, this would suggest that the factor antagonizes the TC-YIK state (anti-TC-YIK).

Starting from the assumption that TC-YIK state is maintained by regulation of TC-YIK-enriched-promoters, we checked, for each TF knock-down, whether TC-YIK-enriched-promoters were more likely to be affected (either up- or down- regulated) compared to a random event. Knock-down of all factors resulted in significantly more TC-YIK-enriched-promoters being perturbed (in either direction) than expected (hypergeometric probability test, Supplementary Table 8), and testing the up- and down-regulated sets separately also showed that for all perturbations significantly more TC-YIK-enriched-promoters were up-regulated and significantly more TC-YIK-enriched-promoters were down-regulated than expected by chance. This suggests that all tested TFs contribute to some extent to the maintenance of the TC-YIK state (Supplementary Table 8, Figure 3B).

Of particular note, *NEUROD1* knock-down led to down-regulation of 50% of the TC-YIK-enriched-promoters, and *ISL1* knock-down led to up-regulation of the most TC-YIK-enriched-promoters compared to the other factors, suggesting that they are pro- and anti-TC-YIK factors respectively (Figure 3B). To examine this in more detail we calculated the ratios of TC-YIK-enriched-promoters to non-enriched-promoters in the up-regulated sets over the down-regulated sets. High ratios correspond to anti-TC-YIK TFs and low ratios correspond to pro-TC-YIK TFs (Figure 3C). To compare these ratios systematically we used Chi-square with Yates correction to test for significant differences (Supplementary Table 8).

Using the above mentioned metric the TC-YIK-enriched factors *MNX1, NEUROD1, SHOX2, PAX4, NROB2, HOPX, RFX6, MLXIPL, GATA4, LMX1A, PAX6, ASCL1* and the non-enriched factors *ATF5, TAF10, HMGA1, TCF25, TAF9, HMGB2, GTF3A* all appear to be pro-TC-YIK (Figure 3C). In the case of *ISL1* and *PROX1* the ratios are shifted in the opposite direction with a higher fraction of up-regulated TC-YIK-enriched-promoters compared to non-enriched-promoters, indicating they act as antagonists to the TC-YIK state (Figure 3C). Interestingly, *MNX1* knock-down led to up-regulation of many non-enriched-promoters (10,483 up vs. 4426 down, ratio = 2.37), and relatively few TC-YIK-enriched-promoters (821 up vs. 1453 down, ratio = 0.57). Thus, *MNX1* is pro-TC-YIK but appears to do this by actively repressing non-enriched-promoters.

### TC-YIK TFs regulate pancreatic genes

Many GO terms were significantly enriched in the up- and down-regulated gene sets, including terms related to pancreatic development and function (Supplementary Table 9). In particular, the following down-regulated gene sets were enriched for the terms “pancreas development” (*ATF5, MNX1, NEUROD1, PAX4, RFX6, SHOX2, TAF9*), “insulin secretion” (*ATF5, GATA4, HOPX, LMX1A, MLXIPL, MNX1, NEUROD1, NROB2, PAX6, RFX6, SHOX2, TAF10, TAF9, TBP*), “cellular response to insulin stimulus” (*ATF5, GATA4, LMX1A, MLXIPL, NEUROD1, NROB2, PAX4, PAX6, RFX6, TAF9, TCF25*), “glycogen biosynthetic process” (*ATF5, HOPX, LMX1A, MNX1, NEUROD1, NROB2*), glycogen catabolic process (*GTF3A, NROB2, SHOX2*), and “glycogen metabolic process” (*HOPX, NEUROD1, NROB2*). While, for the upregulated gene lists, *ISL1* appears to be an antagonist to the pancreatic program with its knockdown leading to up-regulation of a gene set enriched for the terms “glucose homeostasis,” “pancreas development,” “regulation of glucose metabolic process,” “insulin secretion,” “endocrine pancreas development,” “endocrine system development,” and “peptide hormone secretion” (Supplementary Table 9).

In summary, it appears that both enriched and non-enriched factors contribute to the TC-YIK TRN and that, intriguingly, despite *ISL1* and *PROX1* both being enriched in TC-YIK, they seem to be antagonists to the system (Figure 3D).

### Protein-DNA edge mapping by ChIP-seq of *NEUROD1, LMX1A, RFX6*, and *PAX6*

**Figure 4:**
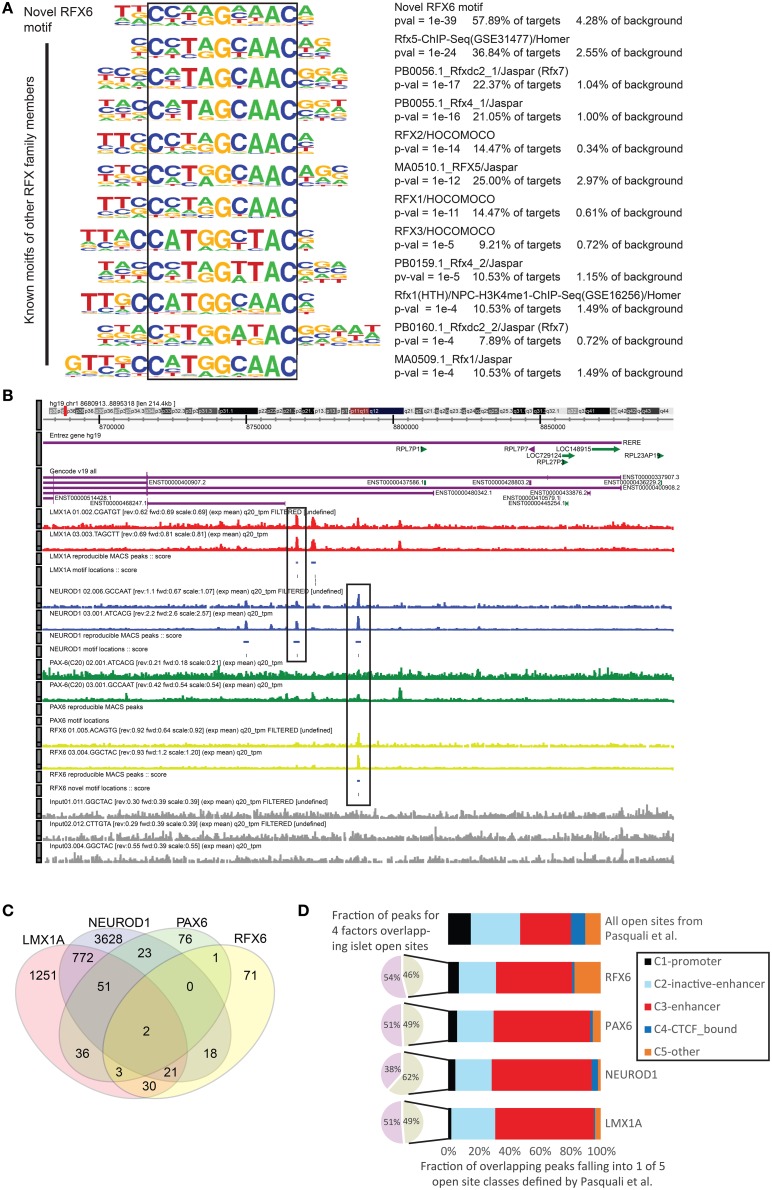
**ChIP-seq analysis of ***NEUROD1***, ***LMX1A***, ***PAX6***, and ***RFX6*** in TC-YIK cells. (A)** Comparison of the novel *RFX6* motif to that of other *RFX* members. Note that it is unlikely that the antibody used (S-15, Santa Cruz) would cross react with any other *RFX* family member as the antibody is raised against a peptide in the unique extended C-terminus of the protein which is not present in any of the other RFX family members. **(B)** ZENBU genome browser (Severin et al., 2014) view showing combinatorial binding of *LMX1A*-*NEUROD1* and *NEUROD1-RFX6* in the first intron of the *RERE* locus. Red, *LMX1A*; Blue, *NEUROD1*; Green, *PAX6*; Yellow, *RFX6*; Gray, input chromatin. **(C)** Venn diagram showing the degree of overlap between the peaks called for the four factors, numbers correspond to count of peaks overlapping by at least 1 base. **(D)** Comparison of the TF ChIP-seq peaks to open chromatin sites identified in human islet cell material by Pasquali et al. (2014).

As the perturbation edges identified above could be either direct or indirect, we next used ChIP-seq data for four of the TC-YIK enriched factors to generate a paired complimentary dataset which would identify the genomic binding sites of the same factor. Integration of these two edge types (KD-CAGE and ChIP-seq) should allow us to discriminate direct from indirect edges. Biological duplicates for each factor were generated and ChIP-seq binding peaks were called relative to input chromatin using MACS (Zhang et al., 2008). We note that the number of peaks called for the same target in different biological replicates varied (NEUROD1: 7195 and 14,949 peaks, LMX1A: 7622 and 7361 peaks, PAX6: 587 and 7866 peaks, RFX6: 960 and 1659 peaks). To be conservative we only used peaks that were called as reproducible with 90% likelihood using the irreproducible discovery rate (Li et al., 2011) method (IDR ≤ 0.1) which yielded 144 RFX6 peaks, 190 PAX6 peaks, 4506 NEUROD1 peaks and 2166 LMX1A peaks. Scanning these peaks for known TFBS motifs using HOMER (Heinz et al., 2010) found significant enrichment for the relevant motifs (NeuroD1/Homer motif was found in 46% of NEUROD1 peaks, 7.4% of background; Lmx1a-mouse/Jaspar–9% of LMX1A peaks, 4.7% of background; PAX6/SwissRegulon–11% of PAX6 peaks, 2.2% of background, Supplementary Figure 3). For *RFX6* there is no known motif; however, the motifs of other *RFX* family members, and in particular *RFX5*, were enriched (37% of RFX6 peaks and 3% of background). *De-novo* motif finding on the *RFX6* ChIP-seq data identified a novel motif that is found in 58% of RFX6 peaks and 4% of background sequences. This motif closely resembles, but is different from, other RFX family motifs (Figure 4A).

Examining the distribution of binding in the genome, we observed that the four factors often bound in combination at the same sites, and seldom bound at promoters. For example in the *RERE* locus we observed co-binding of *NEUROD1* and *LMX1A*, and *NEUROD1* and *RFX6*, respectively, at distinct sites (see boxes in Figure 4B). Genome wide, co-binding of two or more of these enriched factors was common, with more than half of the *RFX6* and *PAX6* sites overlapping a *LMX1A* or *NEUROD1* site (Figure 4C).

Given (1) the paucity of promoter proximal binding of these factors and (2) the ample similarity between TC-YIK cellular program and endocrine program, we compared the binding sites to a map of open chromatin sites in human islet cells. Pasquali et al. (2014) integrated FAIRE-seq, and ChIP-seq of H2A.Z, H3K4me1, H3K4me3, H3K27ac, and CTCF to classify open sites in the genome of human islets as promoters (C1), poised/inactive enhancers (C2), active enhancers (C3), CTCF-bound sites (C4), and other open sites (C5). In our ChIP-seq data, we found that between 46 and 62% of peaks overlapped at least one of these open chromatin sites (this was comparable to the overlap seen by the authors for their own TF ChIP-seq experiments; 48 to 81% for *NKX2.2, PDX1, FOXA2, NKX6.1*, and *MAFB*). For those peaks overlapping the islet cell open sites, we observed enriched binding at active enhancer sites and depletion of promoter sites for all four factors (Figure 4D, Supplementary Table 10), suggesting that these factors primarily work at enhancers.

In support of this observation, both *NEUROD1* and *PAX6* have been reported previously to bind enhancer regions (Andersen et al., 1999; Aota et al., 2003; Scardigli et al., 2003; Inoue et al., 2007; Babu et al., 2008), and a recent *PAX6* ChIP-seq dataset in neuroectoderm cells identified multiple *PAX6* regulated enhancers, and reported that less than 2% of 16,000 *PAX6* peaks are near TSS of coding genes (Bhinge et al., 2014). In the case of *RFX6* there is still little known about its functional targets. Other *RFX* family members have been reported to be bound at enhancers (Reith et al., 1994; Maijgren et al., 2004; Creyghton et al., 2010; Watts et al., 2011), and in the Pasquali *et al*. study an *RFX* motif was over-represented at islet cell enhancer clusters (Pasquali et al., 2014). Intriguingly, *RFX6* had twice as many peaks overlapping class C5 than expected, suggesting that *RFX* binding may be one of the earliest events at opening of sites (Niesen et al., 2005). For *LMX1A*, ours is the first report of its involvement at enhancers.

### Integration of ChIP-seq and KD-CAGE data to identify direct transcriptional targets of TFs

**Figure 5:**
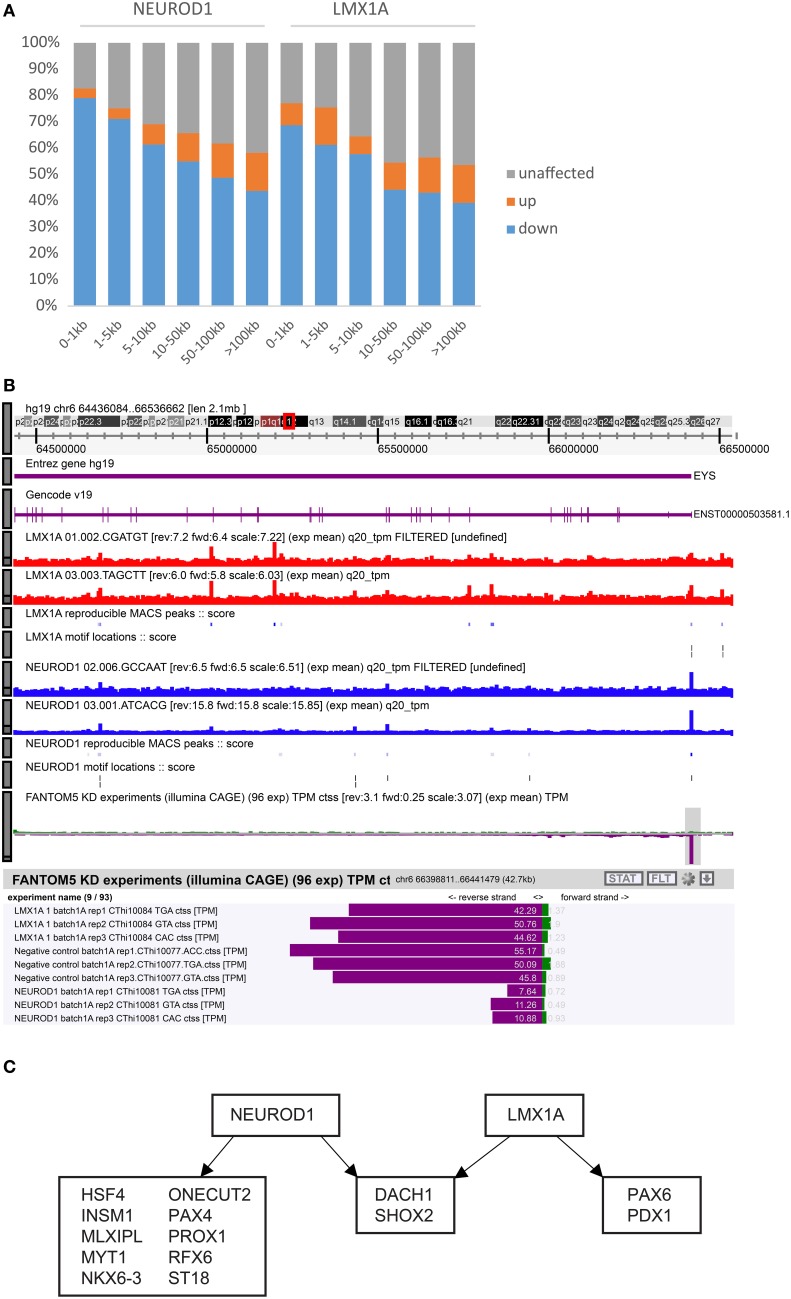
**Integration of KD-CAGE and ChIP-seq to identify direct edges. (A)** Bar graph showing the fractions of up-regulated (orange), down-regulated (blue), and unaffected (gray) TSS in the knock-down of *NEUROD1* or *LMX1A*. Bars correspond to different distance bins from a ChIP-seq peak for the same factor. **(B)** Example of putative non-functional binding of *LMX1A* at the *EYS* locus. Note the presence of multiple *NEUROD1* and *LMX1A* ChIP-seq peaks and relevant motifs, but only the *NEUROD1* knock-down affected *EYS* expression (more examples shown in Supplementary Figure 4). **(C)** Diagram showing TC-YIK enriched transcription factors (from Supplementary Table 4) that are directly regulated by *NEUROD1* or *LMX1A*. To be called a direct target, we require at least one TSS of the target gene to be down-regulated 1.5-fold with a *p*-value of 0.05 and it must be within 50 kb of a ChIP-seq peak for the same factor.

By combining KD-CAGE with ChIP-seq data for *LMX1A, NEUROD1, PAX6*, and *RFX6*, we hoped to identify directly regulated promoters (that is, promoters perturbed in the knock-down experiments that also had matching nearby ChIP-seq signal). In the case of *NEUROD1* and *LMX1A*, we observed that promoters closest to a matching ChIP-seq peak were indeed affected. In particular for *NEUROD1*, almost 80% of promoters within 1 kb of a NeuroD1 ChIP-seq peak were down-regulated and for *LMX1A* almost 70% of promoters within 1 kb of an Lmx1a ChIP-seq peak were down-regulated (Figure 5A). Both cases indicate that these factors work primarily as transcriptional activators. As one moves further away from a ChIP-seq peak the fraction of down-regulated promoters drops, however, even at distances greater than 5 kb (up to 100 kb) from a TSS we observed a higher proportion of down-regulated TSS compared to that seen for those >100 kb away, suggesting that both factors can affect gene expression in *cis* from neighboring enhancer elements (the closer the element, the higher the probability of being affected). Repeating the analysis only using peaks with or without a TFBS motif showed no significant differences in the fractions of TSS likely to be affected. In fact, for the case of *LMX1A* and *NEUROD1* the fraction of perturbed TSS increased at shorter distances relative to a ChIP-seq peak, regardless of whether the ChIP-seq peak overlapped a motif or not (Supplementary Table 11). In the case of *RFX6* and *PAX6*, we observed no such distance-dependent effect, suggesting that either these factors work predominantly via distal sites or that the small number of ChIP-seq peaks observed for these two factors confounded the analysis.

Finally it is worth noting that not all proximal sites appear to be functional. For *NEUROD1* and *LMX1A* respectively, 17 and 18% of the TSSs within 1 kb of a ChIP-seq peak for the same factor were unaffected in the knock-down. An example is shown for the *EYS* locus. ChIP-seq and TFBS predictions support binding of *LMX1A* and *NEUROD1* at the *EYS* promoter, but only *NEUROD1* perturbation affected *EYS* expression levels (Figure 5B; other examples are shown in Supplementary Figure 4).

### Role of *NEUROD1* and *LMX1A* in the TC-YIK TRN

Our original objective had been to integrate KD-CAGE and ChIP-seq to identify directly regulated targets (in this case of *NEUROD1, LMX1A, PAX6*, and *RFX6*). However, based on the results above, we conclude that the majority of binding events happen at enhancers, and only in the case of *NEUROD1* and *LMX1A* where we observed enrichment for perturbed TSS at shorter distances to the TSS can we infer direct promoter mediated edges. For these two factors, we considered TSS that are down-regulated at least 1.5-fold and with a ChIP-seq peak at a distance of less than 50 kb as likely direct targets. This identified 317 and 1543 directly regulated promoters for *LMX1A* and *NEUROD1* respectively (Supplementary Table 12). Finally, to understand the hierarchy of these factors we checked whether they directly regulate any of the other TC-YIK enriched TFs identified in the beginning of the paper. Focusing on the core network (TF-TF) we find that both NEUROD1 and LMX1A directly target 12 and 4 TC-YIK enriched TFs, respectively, but do not directly regulate each other (Figure 5C).

## Conclusion

In this paper we have introduced an experimental strategy to elucidate cell type specific transcriptional regulatory networks. We start by identifying cell type enriched transcription factors (pre-computed lists for all primary cell types available online from the FANTOM web resource (Lizio et al., 2015) http://fantom.gsc.riken.jp/5/) and then use a combination of siRNA perturbation, CAGE and ChIP-seq to identify their direct and indirect targets. This strategy leverages the strengths of both approaches. Application of CAGE to siRNA perturbed samples identifies affected genes and ChIP-seq identifies directly bound targets. We show that ChIP-seq alone is insufficient to discriminate functional from non-functional bound sites, while perturbation approaches alone cannot unequivocally discriminate direct from indirect targets. It is important to precise that we are not questioning the power of ChIP methods in identifying direct and indirect *binding* (Gordan et al., 2009); the novelty of our approach lies in demonstrating that even in the presence of a TF-DNA interaction, *regulation* of target genes can happen only if the site of interaction is functional. This work highlights an important and yet undervalued matter, as in many previous publications researchers have assumed the nearest gene to, or any gene within a fixed distance of, a ChIP-seq peak, is a direct target (Shin et al., 2009; Bottomly et al., 2010; Tallack et al., 2010; Schodel et al., 2011). This is clearly an oversimplification. We have shown that almost a fifth of TSS within 1 kb of a *NEUROD1* or *LMX1A* ChIP-seq peak are unaffected in matching siRNA knock-down. This could mean that these sites are non-functional or that they are cell-context dependent (Osmanbeyoglu et al., 2012; Whitfield et al., 2012).

Aside from exploring this strategy to build TRNs, we have introduced TC-YIK as a model to study transcriptional regulation of pancreatic genes. There is a need for such cell line models, as the majority of viable post mortem islet cell material is used for transplants into diabetic patients, thus pancreatic beta cells for research are difficult to obtain. Moreover, the isolation of pure beta cell populations, the lack of protocols to expand them in culture and the number of cells required to carry out extensive perturbation and chromatin immuno-precipitation experiments are prohibitive. We have shown by CAGE profiling that 85% of the beta cell genes identified by the beta cell gene atlas (Kutlu et al., 2009) are expressed in TC-YIK and that *NEUROD1, LMX1A, PAX6*, and *RFX6* binding sites in TC-YIK are enriched at islet cell active enhancer sites. Furthermore, TC-YIK cells express key transcription factors known to be involved in pancreatic cell development and differentiation, including *NEUROD1, PDX1*, and *FOXA2* (Wang et al., 2002; Itkin-Ansari et al., 2005; Guo et al., 2012). In fact, 33 of the top 42 most TC-YIK enriched TFs are implicated in pancreatic biology. In addition, 33 homolog TFs are expressed in developing mouse pancreas. On this account, we, for the first time, find evidence of *ASCL2, HLF, HSF4, IRF6, IRF8, C11orf9/MYRF*, and *NPAS3* playing a role in pancreatic neuroendocrine gene expression and development. The only two TFs without prior references in the literature or detectable expression in the FANTOM5 mouse pancreatic samples were *SIX3* and *DLX6*, respectively. Despite this, *DLX6* expression has previously been reported in earlier pancreatic stages (E12.5 and E13.5; Gasa et al., 2004). This thorough review shows that the majority of transcription factors with enriched expression in TC-YIK have a role in pancreatic development and thus, TC-YIK is an important cell line model for studying transcriptional regulation of pancreatic gene expression.

Genome-wide expression profiling of the perturbed samples by CAGE revealed multiple insights. The majority of TF knock-downs led to more down-regulated genes than up-regulated ones, suggesting these TFs primarily work as activators, in agreement with the arguments of Hurst et al. (2014). From this logic, we predict *HMGA1, NEUROD1, LMX1A, SHOX2, NROB2, GATA4, RFX6* as likely activators and *MNX1* and *TBP* as likely repressors. Although there is the possibility that a predicted activator is in fact a repressor of an activator and a predicted repressor is an activator of a repressor, we find that both *GATA4* (Rojas et al., 2008) and *LMX1A* (Andersson et al., 2006) have direct evidence as transcriptional activators and *MNX1* (William et al., 2003) has been confirmed as a transcriptional repressor. By incorporating ChIP-seq data we can verify the roles of TFs directly. For both *NEUROD1* and *LMX1A* we show that they work as direct transcriptional activators. This clarifies the role of *NEUROD1* as a previous work reported it as both a transcriptional repressor and activator (Itkin-Ansari et al., 2005). Integration of the CAGE and ChIP-seq data clearly shows that >75% of TSS proximal to *NEUROD1* are down-regulated in *NEUROD1* knock-down (Figure 5A). In the previous work by Itkin-Ansari et al. the authors used perturbation (over-expression) alone and assumed *SST* down-regulation upon *NEUROD1* over-expression indicated it was a target that was directly transcriptionally repressed; we think it is more likely that *NEUROD1* indirectly antagonizes *SST* expression via other pancreatic TFs. This highlights the value of using both perturbation and ChIP-seq approaches.

In terms of what the application of our strategy to TC-YIK has told us about pancreatic gene expression, and the hierarchy of TFs, firstly we have shown that not only enriched (*MNX1, NEUROD1, SHOX2, PAX4, NROB2, HOPX, RFX6, MLXIPL, GATA4, LMX1A, PAX6, ASCL1*) but also non-enriched factors (*ATF5, TAF10, HMGA1, TCF25, TAF9, HMGB2, GTF3A*) contribute to the maintenance of the TC-YIK state. It is thus important to consider housekeeping TFs, too, when building cell-specific TRNs since they often work cooperatively with state specific factors (Ravasi et al., 2010). Our analysis also identified *ISL1* and *PROX1* as likely antagonists to the state. It may be that these antagonists help maintain a stem/progenitor like state (Wang et al., 2005; Eberhardt et al., 2006). We show that *NEUROD1* and *LMX1A* are both directly activating multiple other pancreatic TFs, and that based on our data they do not directly regulate each other (Figure 5C).

Finally, building cell-type-specific TRNs will require further work and integration of newer data types. In the case of *RFX6* and *PAX6* we made no predictions of their direct targets as there were few peaks bound at promoter regions and there was no enrichment for perturbed TSS near these peaks. This could be due to lower quality or less efficient antibodies used for the two factors, or could reflect lower expression levels compared to the other factors. Despite this, for all four factors (including the higher quality *NEUROD1* and *LMX1A* experiments) the majority of peaks were at putative enhancer regions. In conclusion, mammalian TRN models will need to incorporate distal regulatory elements as well, as proximal elements. To address this issue in the future we will need to use protocols such as ChIA-PET (Fullwood et al., 2009) and HiC (Dixon et al., 2012) to link distal elements with the TSS that they regulate. We believe that such chromatin conformation methods combined with KD-CAGE and ChIP-seq have the potential to identify gold standard regulatory events at both promoters and enhancers, and are key to understanding how each cell type is wired.

## Methods

### Selection of transcription factors significantly enriched in TC-YIK for siRNA knock down

A pre-computed list of TFs with enriched expression in TC-YIK was downloaded from FANTOM5's sample browser SSTAR [direct link: http://fantom.gsc.riken.jp/5/sstar/FF:10589-108D4, see FANTOM web resource (Lizio et al., 2015)]. Enrichment is based on expression in the sample compared to the median expression across all samples in the FANTOM5 collection. The enrichment score is defined as log10[(expression in TC-YIK + 1)/(median expression in FANTOM5+1)]. The top 33 genes with enriched expression in TC-YIK were targeted for siRNA knock-down using stealth siRNAs from Invitrogen. As a comparison we also targeted a set of 8 non enriched TFs (*TAF9, TAF10, ATF5, GTF3A, TCF25, TBP, HMGA1, HMGB2*) that were expressed in TC-YIK at similar levels. In addition to these TFs, six target genes (*INS, CHGA, GHRL, GCK, GAST, TTR*) and five additional target TF genes where we were unable to find effective siRNAs (*ASCL2, CBFA2T2, CDX2, INSM1, TFAP2A*) were also added to the set. The combined set was used for systematic siRNA KD in triplicate of one factor at a time followed by qRT-PCR measurements of the perturbed genes in a Matrix RNAi design as described in Tomaru et al. (2009). siRNA sequences, knock-down efficiency and primers used in qRT-PCR are provided in Supplementary Table 9.

### Cell culture

TC-YIK (Ichimura et al., 1991; Human cervical cancer) cells were provided by RIKEN BRC (Cell no: RCB0443). Cells were grown in RPMI1640 (GIBCO), 10% fetal bovine serum (CCB), 1% penicillin/streptomycin (Wako). TC-YIK cells were incubated at 37°C in a humidified 5% CO2 incubator.

### Genome-wide KD-CAGE

KD experiments followed by CAGE were profiled (see below) to obtain genome-wide promoter activities. Of the 41 most enriched TFs that were selected for Matrix RNAi, 15 among the most perturbed and all 8 non-enriched genes were chosen for siRNA transfection followed by CAGE. The 15 enriched TFs targeted for CAGE analysis were selected in a semi-random fashion that favored TFs that affected insulin expression in the qRT-PCR results (Figure 2). *NEUROD1, DACH1, RFX6, ASCL1, PAX6, MNX1, HOPX, MLXIPL, LMX1A, SHOX2, GATA4*, and *PAX4* knock-down significantly reduced *INS* transcript levels. *PROX1, NR0B2*, and *ISL1* were selected based on their reported roles in pancreatic biology as putative repressors, rather than their effect on *INS* levels. Experiments were carried out in biological triplicate, and scrambled siRNA samples were prepared as negative control. While the KD method has been previously described (Vitezic et al., 2010), we used a new variant of CAGE developed for the Illumina Hiseq 2500 called nAnT-iCAGE (Murata et al., 2014). Briefly, 5 μg of RNA was used for each sample and libraries were combined in 8-plex using different barcodes. Tags were de-multiplexed and mapped to the human genome (hg19) using BWA (Li and Durbin, 2010), yielding an average of 8.9 M mapped counts per sample (map quality > 20). Expression tables were made by counting the numbers of mapped tags falling under the 184,827 robust CAGE peaks regions identified in FANTOM5 (Forrest et al., 2014). Differential expressed promoters in TF knock-downs vs scrambled controls were identified using edgeR (Robinson et al., 2010) with a significance threshold of 0.05.

### Chromatin immunoprecipitation assay

Chromatin was prepared and immunoprecipitation carried out as described previously (Kubosaki et al., 2009).

List of antibodies used in the ChIP-seq experiments: *LMX1A* [LMX1A (C-17), sc-54273X Santa Cruz], *NEUROD1* [Neuro D (G-20), sc-1086X Santa Cruz], *RFX6* [RFX6 (S-15), sc-169145X Santa Cruz], and *PAX6* [Anti Pax-6 (C-20], Human (Goat), sc-7750 X Santa Cruz]. Note to readers, the following antibodies were also tried but failed in ChIP-seq: [Santa Cruz: Anti ISL1 (K-20) sc-23590X; Anti PAX6 (AD2.38) sc-32766X; Anti Dlx-6 (G-20) sc-18154; Anti HB9 (H-20) sc-22542; Anti DLX6 (C-20) sc-18155; Anti PDX-1 (A-17) sc-14664 X; and Abnova: Anti ISL1 (H00003670-M05)].

All experiments were carried out as biological duplicates. Immunoprecipitated and input chromatin samples were incorporated into 4-plex ChIP-seq libraries using the NEBnext kit (New England Biolabs). Libraries were labeled with a 6 bp barcode and then pooled to be sequenced on Illumina HiSeq2000.

Sequencing results were mapped to the human genome (hg19) using BWA software (Li and Durbin, 2010) providing an average of ~180 M mapped tags per lane (or, alternatively, ~45 M per sample), with a mapping rate of >96%. After mapping we performed peak calling using MACS software (Zhang et al., 2008) with the recommended default parameter settings for point binding type of events [mfold = (Refai et al., 2005; Tompa et al., 2005), bandwidth = 300]. We additionally used Irreproducible Discovery Rate analysis (Li et al., 2011), to identify reproducible peaks which were used for downstream analysis.

### Motif enrichment analysis

We used HOMER software for de-novo motif discovery (Heinz et al., 2010), as well as to calculate over-representation of known motifs. Known motifs provided with HOMER (v4.6, 3-29-2014) were expanded by importing all known *NEUROD1, LMX1A, PAX6*, and *RFX* motifs from SwissRegulon (Pachkov et al., 2007), JASPAR (Bryne et al., 2008), UniPROBE (Newburger and Bulyk, 2009), and HOCOMOCO (Kulakovskiy et al., 2013), into HOMER before carrying out the scan. We used the function *findMotifsGenome.pl* to discover motifs in all reproducible peaks for each factor (genomic regions from hg19) with the option “–mask” to filter out bindings on repeats. The target sequences are the regions under the peaks and the background regions are randomly sampled sequences from the genome (Hg19) with similar GC content as the target sequences.

### Gene ontology enrichment analysis

The R Bioconductor GOstats package (Falcon and Gentleman, 2007) was used to obtain gene ontology enrichment scores. For the ChIP-seq GO analysis was performed on bound TSSs, while for the CAGE KD experiments, the up- and down-regulated genes were analyzed separately. For both analyses, all genes expressed in TC-YIK (>1 TPM) were used as the background.

### Data access

This work is part of the FANTOM5 project. Data download, genomic tools and co-published manuscripts have been summarized at http://fantom.gsc.riken.jp/5/. A ZENBU genome browser view displaying TC-YIK related expression data can be accessed at this URL: http://fantom.gsc.riken.jp/zenbu/gLyphs/#config=e3YeqamiJBWhbPgPq59ubD;loc=hg19::chr14:93349815.93441266 [Reviewer username: lizio2014-review@riken.jp, password: lizio2014 (note: if problems after logging in, re-enter the URL and try again. Password will be removed at publication)]. All sequencing data used in this study has been deposited to DDBJ Read Archive (http://www.ddbj.nig.ac.jp/) with accession number DRA002420 (CAGE data) and DRA002468 (ChIP-seq data). CAGE expression profiles and enrichment of TFs for TC-YIK cell line are part of the FANTOM5 main data set. siRNA perturbations, CAGE-KD, and ChIP-seq experiments were generated separately for this study. Additional material can be found at the following URL (http://fantom.gsc.riken.jp/5/suppl/Lizio_et_al_2014/?cultureKey=&q=5/suppl/Lizio_et_al_2014 Reviewer username: m.lizio, password: m.lizio).

## Author contributions

AF designed the study and wrote the manuscript; ML carried out all bioinformatics analyses and wrote the manuscript; YI carried out the siRNA perturbations, qRT-PCR and chromatin immuno-precipitation experiments with help from AK; MI provided the CAGE libraries; TL and AH mapped the CAGE data; YN provided the TC-YIK cell line; JS helped with visualization in ZENBU; HK contributed to the ChIP-seq analysis and provided the set of CAGE peaks; HS, HK, PC, YH, and AF supervised the project.

## Funding

FANTOM5 was made possible by the following grants: Research Grant for RIKEN Omics Science Center from MEXT to Yoshihide Hayashizaki; Grant of the Innovative Cell Biology by Innovative Technology (Cell Innovation Program) from the MEXT, Japan to Yoshihide Hayashizaki; Research Grant from MEXT to the RIKEN Center for Life Science Technologies; Research Grant to RIKEN Preventive Medicine and Diagnosis Innovation Program from MEXT to YH. We thank Michiel de Hoon for proofreading the manuscript. We would also like to thank RIKEN BRC for providing the TC-YIK cell line samples and thank GeNAS for data production. ARRF is supported by a Senior Cancer Research Fellowship from the Cancer Research Trust and funds raised by the MACA Ride to Conquer Cancer.

### Conflict of interest statement

The authors declare that the research was conducted in the absence of any commercial or financial relationships that could be construed as a potential conflict of interest.

## Supplementary material

Supplementary Figure 1 **Homolog TF genes expressed in mouse pancreas development series**. CAGE expression profiles for 33 of the 42 human homolog TC-YIK-enriched TFs. Only TFs with expression above 1TPM for at least one developmental stage are shown. On the x-axis are developmental stages, from E14 until adult state. The y-axis shows expression levels (normalized TPM).

Supplementary Figure 2 **CAGE KD and qRT-PCR KD comparison**. Plots for 23 transcription factors matched in both CAGE and qRT-PCR. Fold changes largely agree between technologies. Each dot represents the fold change value of a target gene among the pool of 52 perturbed genes in the matrix RNAi pilot study.

Supplementary Figure 3 **HOMER Motif scan summary**. Enrichment of relevant known motif and top novel motif is shown for *NEUROD1, LMX1A, PAX6*, and *RFX6*. Expanded results are available online at (http://fantom.gsc.riken.jp/5/suppl/Lizio_et_al_2014).

Supplementary Figure 4 **ZENBU genome browser views showing integration of CAGE and ChIP-seq profiles for LMX1A and NEUROD1. (A)** SYT4 and PLK4 loci have proximal binding of both factors and are affected in both of the knock-downs. **(B)** GPD2 and RSRC1 loci have proximal binding of both factors but are affected in both the knock-downs. **(C)** PROX1 and ID4 have proximal binding of both factors but only the knock-down of NEUROD1 affects expression.

Supplementary Table 1 **Human islet cell enriched transcripts**. Detection of human islet cell enriched transcripts from the beta cell gene atlas (Kutlu et al., 2009) in TC-YIK.

Supplementary Table 2 **Rat alpha and beta cell enriched transcripts**. Detection of human orthologs of rat alpha and beta cell enriched transcripts from the beta cell gene atlas (Kutlu et al., 2009) in TC-YIK.

Supplementary Table 3 **Extended main Table 2**. TFs enriched in TC-YIK and their putative function in pancreas.

Supplementary Table 4 **siRNAs and primers used in this study**.

Supplementary Table 5 **Matrix RNAi results**. Pilot study of systematic knock-down and qRT-PCR expression measurements for TC-YIK enriched transcription factors.

Supplementary Table 6 **Affected targets and in/out degree**. Summary of the matrix RNAi study: numbers of affected targets, in- and out-degree and effects on *INS* gene.

Supplementary Table 7 **Promoters perturbed by TF knockdown**. List of promoters detected by edgeR in KD-CAGE sets (*p*-value of 0.05, 1.5FC).

Supplementary Table 8 **Summary of affected promoters in CAGE KD**. Numbers of differentially expressed promoters in CAGE KD and ratios of affected TC-YIK enriched promoters.

Supplementary Table 9 **Gene ontology enrichment of perturbed genes**. GO enrichment analysis for CAGE KD differentially expressed promoters (split in up- and down- regulated).

Supplementary Table 10 **Overlap with open chromatin regions**. Overlap of TC-YIC ChIP-seq peaks and C1-C5 open chromatin regions as defined in Pasquali et al. (2014).

Supplementary Table 11 **ChIP-seq- CAGE integration**. Relationship between distance from ChIP-seq peak and perturbation in CAGE, for peaks (all, +motif, −motif).

Supplementary Table 12 **Direct targets of NEUROD1 and LMX1A**. TSS that are down-regulated 1.5-fold, *p*-value of 0.05 and within 50 kb of a ChIP-seq peak for the same factor.
