# Supplementary figures and images for: Mapping Mammalian Cell-type-specific Transcriptional Regulatory Networks Using KD-CAGE and ChIP-seq Data in the TC-YIK Cell Line
Source: Front Genet. 2015 Nov 18;6:331. doi: 10.3389/fgene.2015.00331 (PMC4650373; doi:10.3389/fgene.2015.00331)

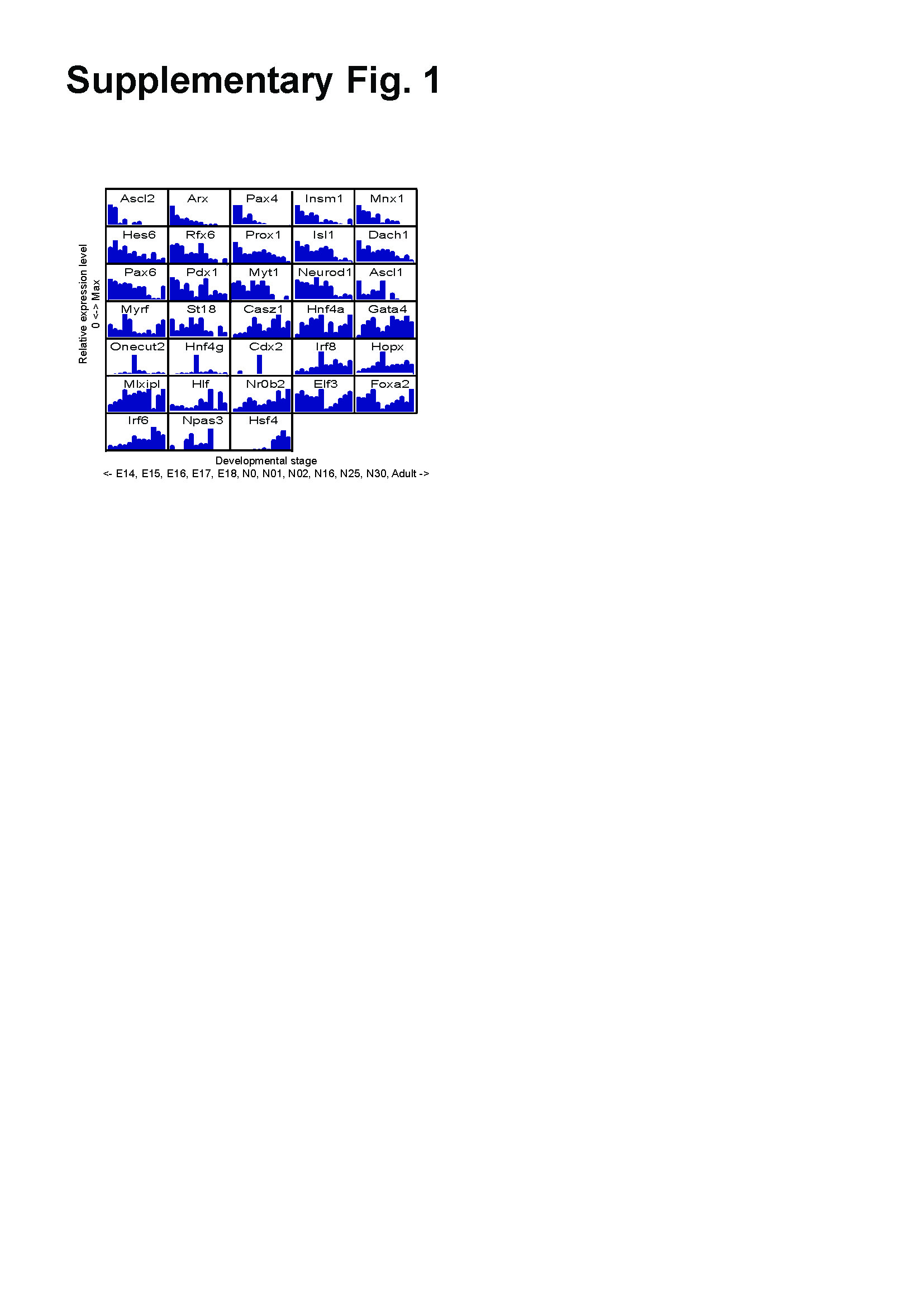

Supplement: Supplementary Figure 1 — Homolog TF genes expressed in mouse pancreas development series. CAGE expression profiles for 33 of the 42 human homolog TC-YIK-enriched TFs. Only TFs with expression above 1TPM for at least one developmental stage are shown. On the x-axis are developmental stages, from E14 until adult state. The y-axis shows expression levels (normalized TPM). [file Image1.JPEG]

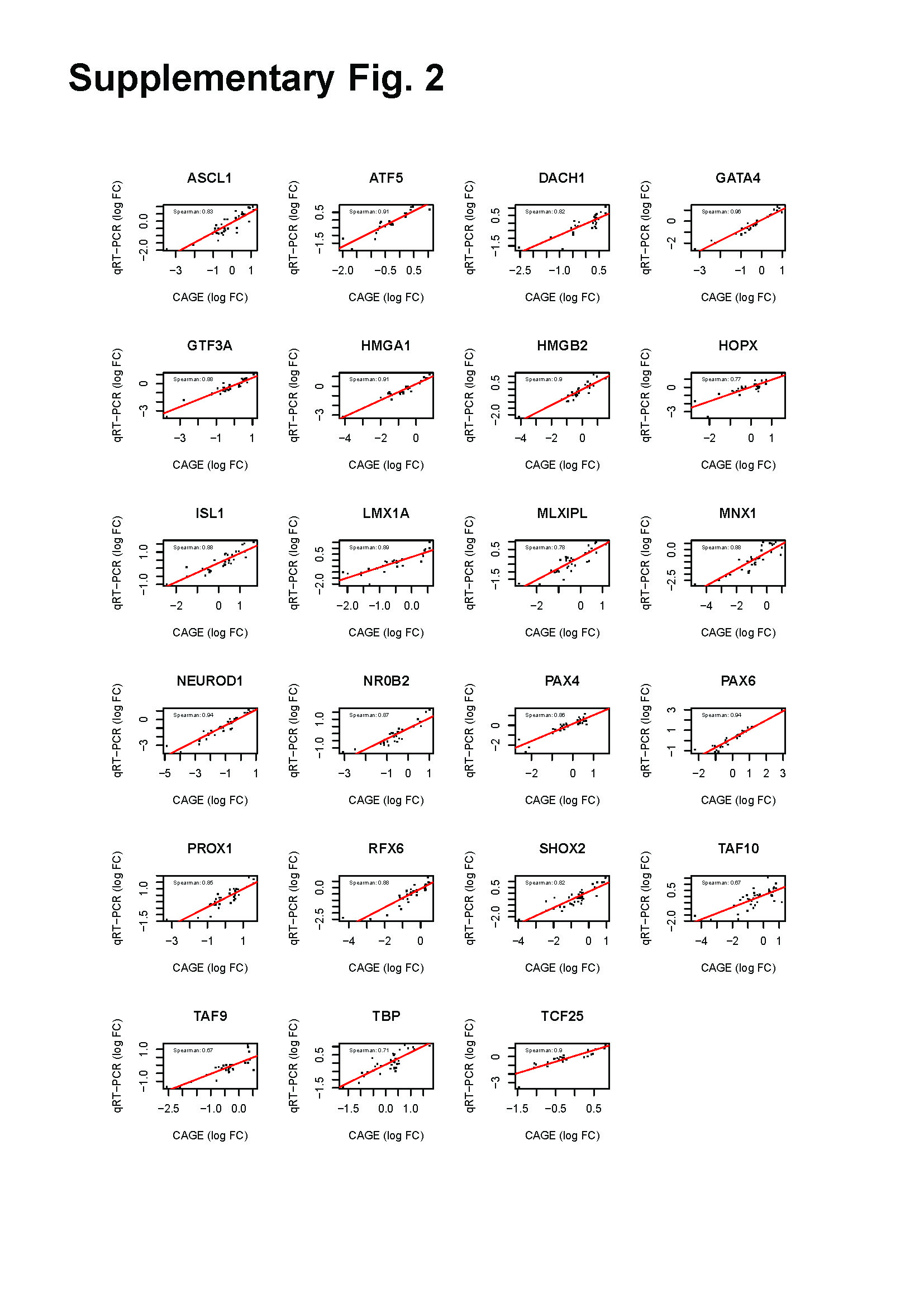

Supplement: Supplementary Figure 2 — CAGE KD and qRT-PCR KD comparison. Plots for 23 transcription factors matched in both CAGE and qRT-PCR. Fold changes largely agree between technologies. Each dot represents the fold change value of a target gene among the pool of 52 perturbed genes in the matrix RNAi pilot study. [file Image2.JPEG]

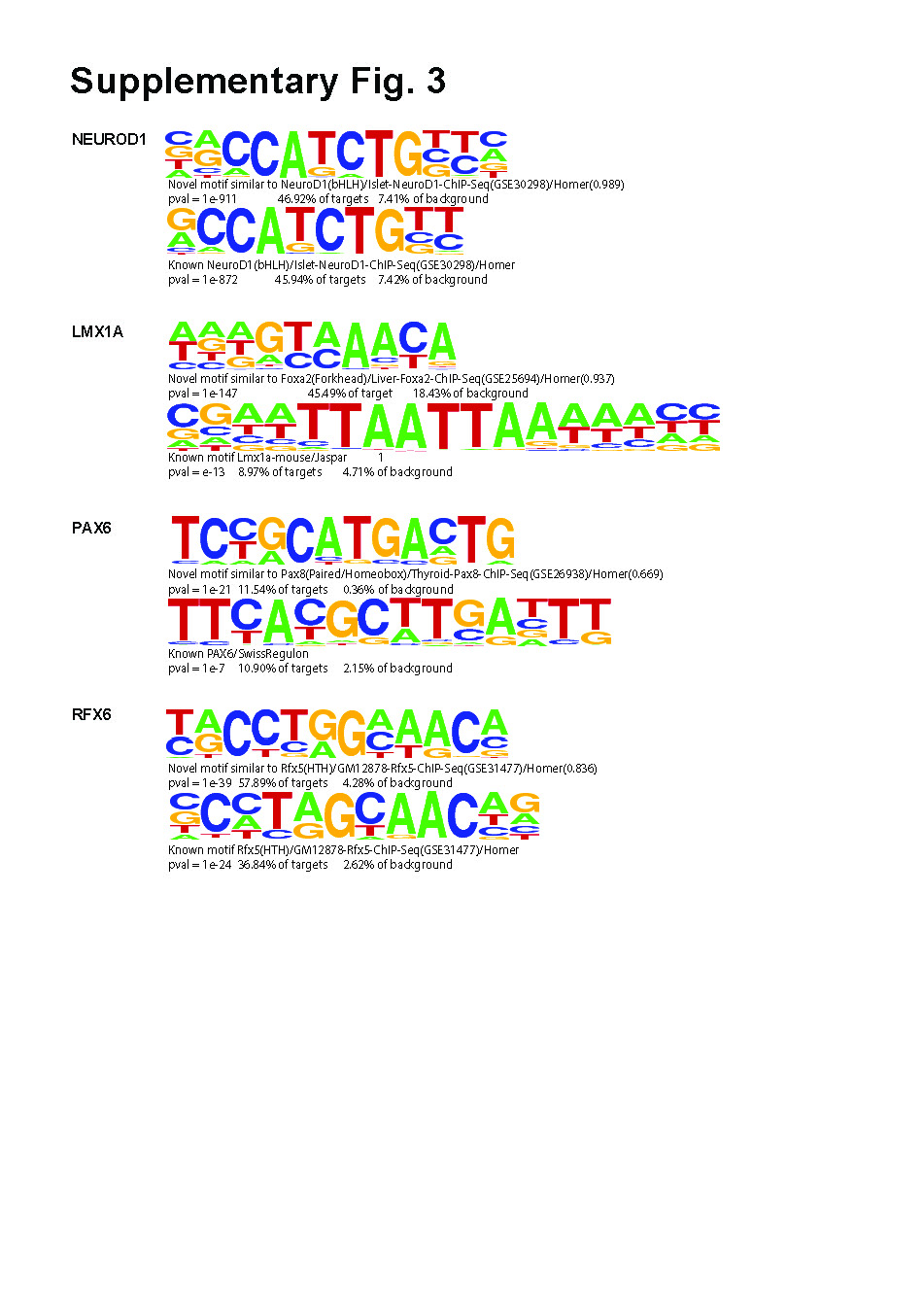

Supplement: Supplementary Figure 3 — HOMER Motif scan summary. Enrichment of relevant known motif and top novel motif is shown for NEUROD1, LMX1A, PAX6, and RFX6. Expanded results are available online at (http://fantom.gsc.riken.jp/5/suppl/Lizio_et_al_2014). [file Image3.JPEG]

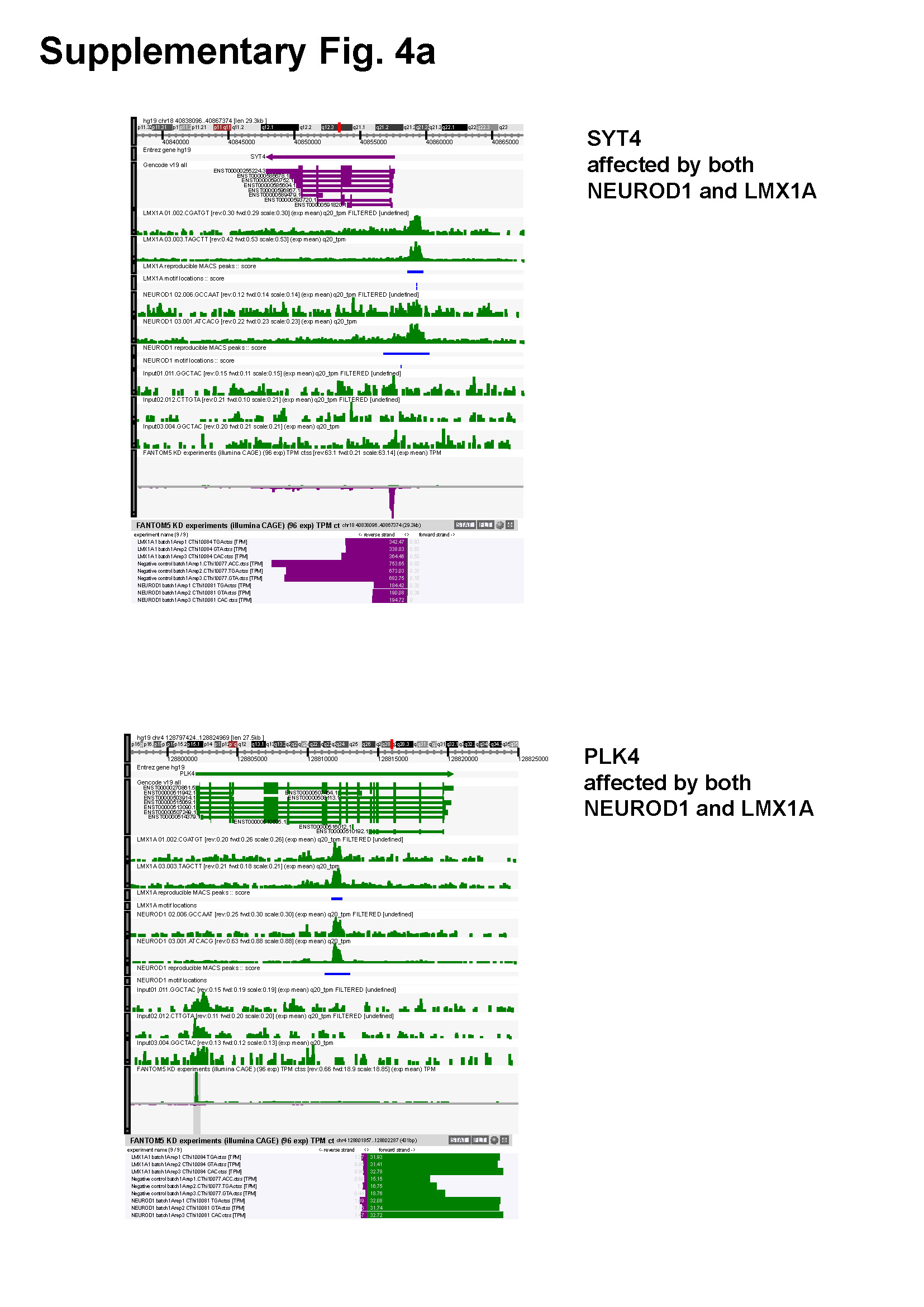

Supplement: Supplementary Figure 4 — ZENBU genome browser views showing integration of CAGE and ChIP-seq profiles for LMX1A and NEUROD1. (A) SYT4 and PLK4 loci have proximal binding of both factors and are affected in both of the knock-downs. (B) GPD2 and RSRC1 loci have proximal binding of both factors but are affected in both the knock-downs. (C) PROX1 and ID4 have proximal binding of both factors but only the knock-down of NEUROD1 affects expression. [file Image4.JPEG]

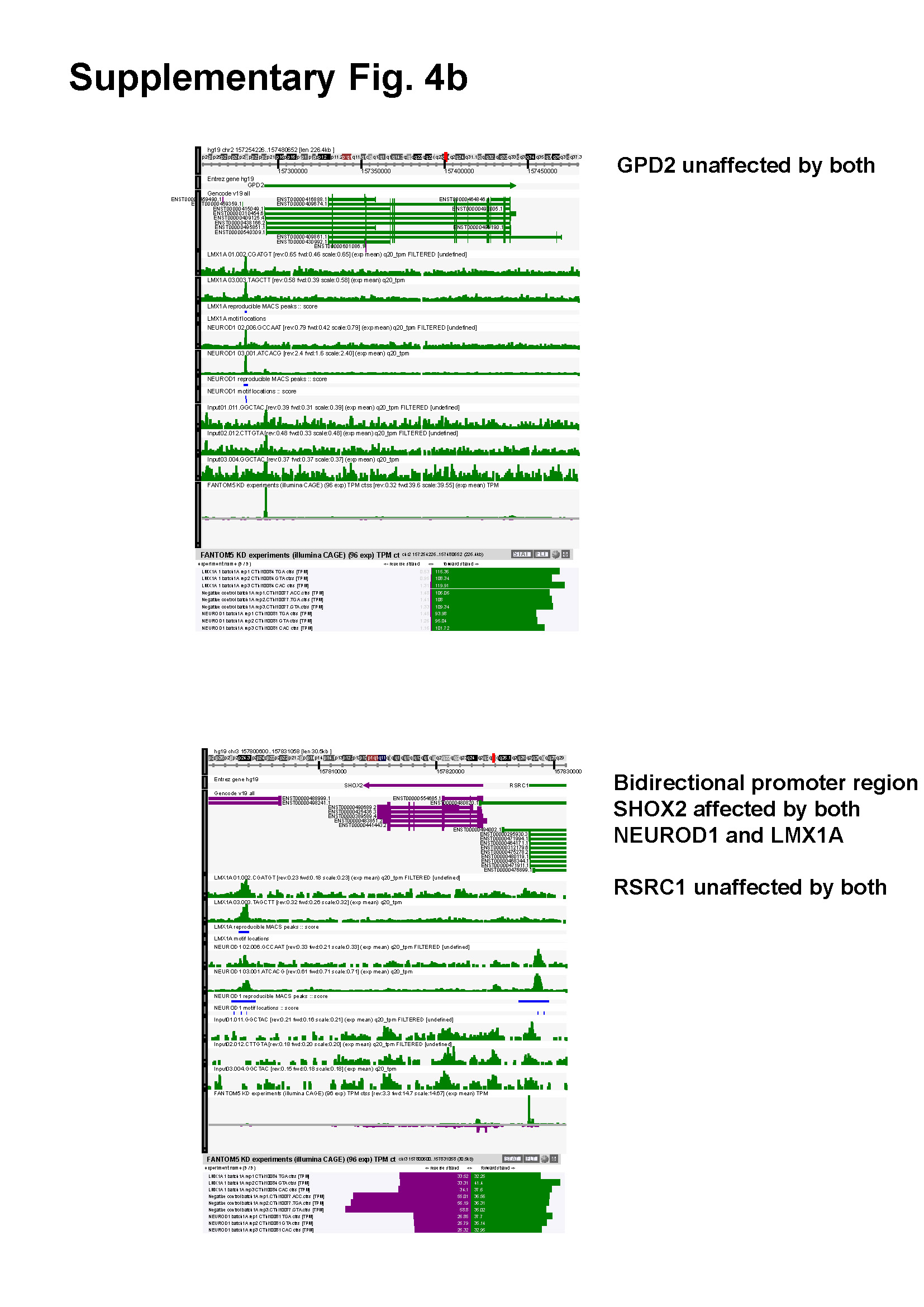

Supplement: Supplementary file 5 [file Image5.JPEG]

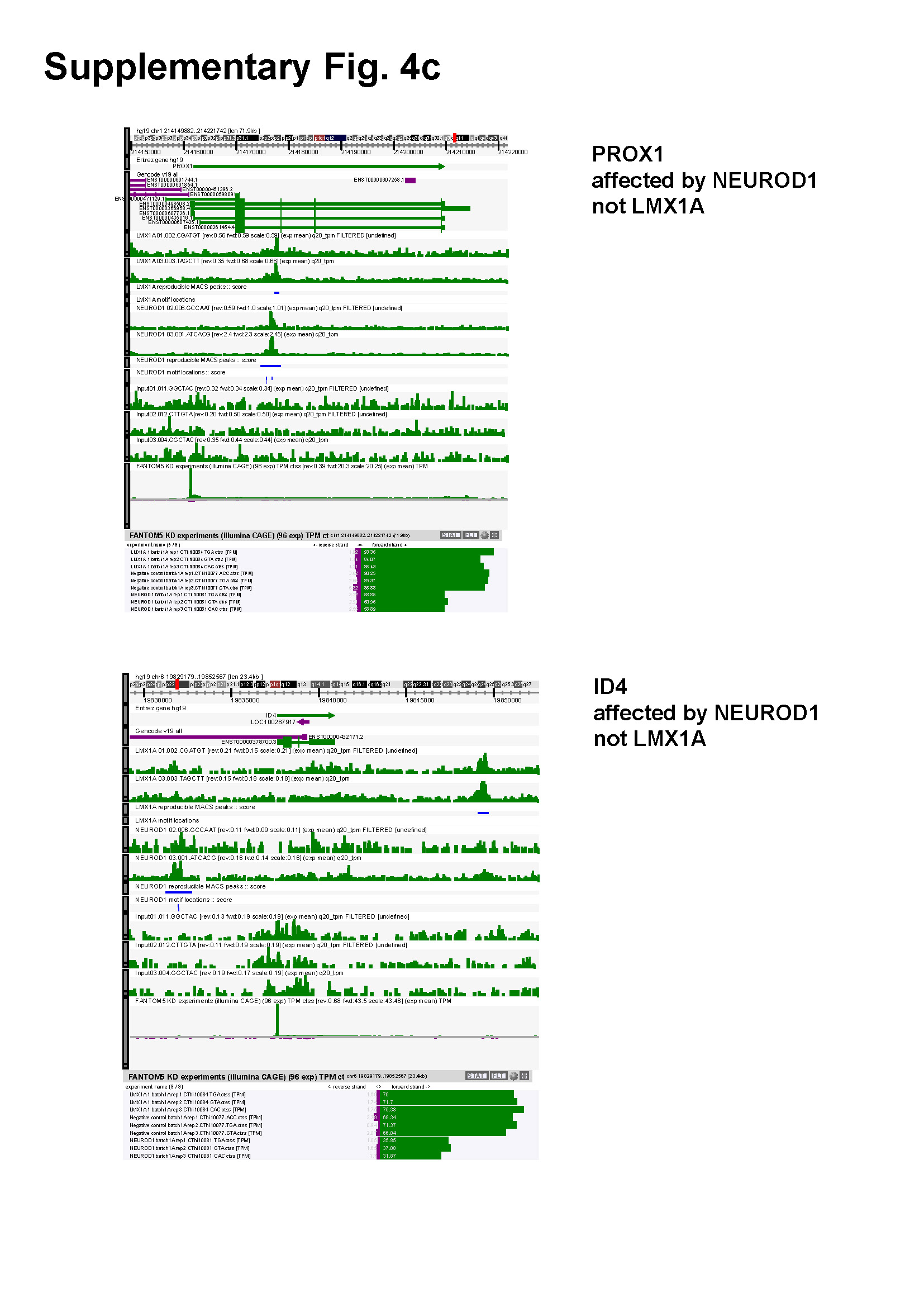

Supplement: Supplementary file 6 [file Image6.JPEG]
